# Supplementary material for: Fatty Acid Unsaturation Degree of Plasma Exosomes in Colorectal Cancer Patients: A Promising Biomarker
Source: Int J Mol Sci. 2021 May 11;22(10):5060. doi: 10.3390/ijms22105060 (PMC8151919; doi:10.3390/ijms22105060)
Supplement: Supplementary file 1 [file ijms-22-05060-s001.zip › ijms-1182243-Supplementary.pdf]

International Journal of *Molecular Sciences*

MDPI

Article Supplementary Data

Fatty acid unsaturation degree of plasma exosomes in colorectal cancer patients: a promising biomarker.

Joan Bestard-Escalas[1,2], Rebeca Reigada[1,2], José Reyes[1,3], Paloma de la Torre[1,4], Gerhard Liebisch[5], and Gwendolyn Barceló-Coblijn[2,*]

[1] Health Research Institute of the Balearic Islands (IdISBa), 07120 Palma, Spain; juan.bestard@uclouvain.be (J.B.-E.) rebeca.reigada@ssib.es (R.R.); jose.reyes@hcin.es (J.R.); paloma.delatorre@ssib.es (P.d.l.T.)
[2] Research Unit, University Hospital Son Espases, 07120 Palma, Spain
[3] Gastroenterology Unit, Hospital Comarcal de Inca, 07300, Inca, Balearic Islands, Spain
[4] Gastroenterology Department, University Hospital Son Espases, 07120 Palma, Spain
[5] Institute of Clinical Chemistry and Laboratory Medicine, University Hospital Regensburg, 93042 Regensburg, Germany; Gerhard.Liebisch@klinik.uni-regensburg.de
* Correspondence: gwendolyn.barcelo@ssib.es; Tel.: +34-871-205000 (ext. 66300)
† Current address: Université Catholique de Louvain, 1200 Brussels, Belgium; juan.bestard@uclouvain.be

Keywords: exosomes; lipidome; colorectal cancer; monounsaturated fatty acids; polyunsaturated fatty acids.

Citation: Bestard-Escalas, J.; Reigada, R.; Reyes, J.; de la Torre, P.; Liebisch, G.; Barceló-Coblijn, G. Fatty Acid Unsaturation Degree of Plasma Exosomes in Colorectal Cancer Patients: A Promising Biomarker. *Int. J. Mol. Sci.* **2021**, *22*, x. https://doi.org/10.3390/xxxxx

Academic Editor: Firstname Last-name
Received: date
Accepted: date
Published: date

Publisher's Note: MDPI stays neutral with regard to jurisdictional claims in published maps and institutional affiliations.

Copyright: © 2021 by the authors. Submitted for possible open access publication under the terms and conditions of the Creative Commons Attribution (CC BY) license (http://creativecommons.org/licenses/by/4.0/).

Supplementary Table 1. Clinical information of the patients participating in the study.

**Healthy Patients Group**	
**Sex**	**Age**
M	37
F	37
F	39
F	39
F	44
F	49
M	50
F	51
F	52
F	59
M	61
F	62
F	66
F	69

**Hyperplastic polyps**		
**Sex**	**Age**	**Location of the lession**
M	43	Transverse
F	45	Transverse, descendent
M	52	Transverse
M	75	Descendent
M	83	Ascendent

**Adenomatous Polyps Group**				
**Sex**	**Age**	**Location of the lession**	**Histological details [a]**	**Nº of polyps**
F	54	Sigma	TV	1
M	56	Ascendent	Tubular	6
F	57	Descendent	Tubular	>20
M	58	Rectum	Tubular	>20
M	61	Ascendent	TV	4
M	61	Sigma	TV	3
F	61	Descendent	Tubular	1
M	63	Rectum	TV	1
M	65	Transverse	Tubular	3
F	65	Sigma	?	1
M	67	Transverse	TV	2
F	67	Ascendent-cecum	Tubular	2
F	68	Tranverse	TV	1
M	74	Rectum	TV with ADC fragments	3
M	78	Ascendent	Tubular	-
F	82	Cecum	Without HGD	3

ç

**Invasive neoplasia – Colorectal Cancer**			
**Sex**	**Age**	**Location**	**Anatomic stage/ TNM classification** [b]
M	59	Rectum	T_3 N_0-N_1
F	78	Sigma	T_3 N_{2a}
F	65	Sigma	T_3 N_0
F	58	Rectum	T_4
F	66	Rectum	T_3 N_0 M_x
M	72	Sigma	T_1 N_0
F	60	Sigma	T_2
M	66	Sigmoide	T_2 N_0
M	47	Sigma	T_4 M_2
M	53	Ascendent	T_4 N_0
F	77	Sigma	T_2 N_0
F	65	Sigma-descendent	T_3 N_0b M_{1a}
F	54	Descendent	T_4 N_0
M	74	Splenicus	T_3 N_0
M	70	Rectum	T_3 N_0
M	71	Descendent	T_3 N_{1b}
M	54	Rectum	T_3 N_3
M	69	Sigma	T_3 N_{1b}
M	87	Rectum	T_3 N_0

**Hereditary CRC**	
**Sex**	**Age**
M	29
F	52
M	41
F	36
M	32
F	46
M	19
M	24
M	43

[(a)] Adenomas are divided into three subtypes depending on the percentage of villous component into: (1) tubular (<25%), (2) tubulovillous (TV, 25-75%), and (3) villous (>75%). Villous adenomas are associated more often with larger adenomas and more severe degrees of dysplasia. HGD: High Grade Dysplasia.

[(b)] The TNM Classification of Malignant Tumors (TNM) is an alphanumeric classification of the cancer stage. T describes the size of the primary tumor and whether it has invaded nearby tissue, N describes nearby (regional) lymph nodes involved, M describes distant metastasis (spread of cancer from one part of the body to another).

Supplementary Table 2. Phospholipid and Sphingolipid lipid composition of plasma EV.

	Healthy		HP		AD		Neo		Her	
	Mean	SEM	Mean	SEM	Mean	SEM	Mean	SEM	Mean	SEM
PC	**36.7**	1.3	**45.0**	6.3	**48.0**	3.3[a]	**46.9**	2.1	**53.0**	2.4[aa]
SM	**33.5**	1.8	**25.1**	3.0	**28.5**	2.5	**28.5**	1.5	**23.2**	1.2 [a]
Cer	**3.0**	0.3	**2.6**	0.2	**2.7**	0.3	**3.4**	0.2	**2.3**	0.2
PE	**7.7**	0.7	**7.5**	1.1	**5.8**	0.7	**7.2**	0.6	**5.7**	0.3
PS	**1.4**	0.1	**1.8**	0.3	**2.0**	0.2	**1.8**	0.1	**2.3**	0.2[aa]
PI	**14.9**	1.7	**15.8**	2.9	**10.8**	1.0	**10.1**	0.7	**11.8**	1.7
LPC	**2.8**	0.2	**2.1**	0.2	**2.1**	0.3	**2.1**	0.2	**1.6**	0.1[a]

Values are expressed as a percentage of total membrane lipid (mole %) and represent the mean ± SEM, n=13 for healthy group, n=5 for HP group, n=16 for AD patients, n=9 for patients for Her patients, and n=19 for Neo patients. Statistical differences were assessed by one-way ANOVA followed by a Bonferroni post-test. a $p < 0.05$, aa $p < 0.01$, Primary vs HP, AD, Neo or Her; Abbreviations: AD: adenomatous polyps; Cer: ceramide; Her: Hereditary CRC; HP: hyperplastic polyps; Neo: Neoplastic lesion (CRC); PC: phosphatidylcholine; PE: phosphatidylethanolamine; PI: phosphatidylinositol; SM: sphingomyelin.

Supplementary Table 3. Phospholipid and Sphingolipid species composition of plasma EVs.

	**Phosphatidylcholine species**									
	Healthy		HP		AD		Neo		Her	
	Mean	SEM	Mean	SEM	Mean	SEM	Mean	SEM	Mean	SEM
PC 30:0	**1.7**	0.1	**1.5**	0.4	**1.0**	0.2[aa]	**1.0**	0.1[aa]	**0.8**	0.1[aa]
PC 32:2	**0.2**	0.0	**0.2**	0.0	**0.2**	0.0	**0.2**	0.0[a]	**0.2**	0.0
PC 32:1	**2.0**	0.2	**1.8**	0.3	**1.5**	0.1	**1.3**	0.1[a]	**1.2**	0.2[a]
PC 32:0	**5.5**	0.5	**3.6**	0.7	**3.8**	0.6	**4.2**	0.5	**2.1**	0.3[aa]
PC 34:3	**0.6**	0.0	**0.7**	0.1	**0.7**	0.1	**0.7**	0.0	**0.9**	0.1[a]
PC 34:2	**14.0**	0.8	**17.5**	3.0	**18.2**	1.3	**20.0**	1.0[a]	**23.8**	2.1[aaa]
PC 34:1	**32.5**	1.2	**28.9**	2.4	**26.0**	2.0	**26.1**	1.4	**20.5**	2.2[aaa]
PC 34:0	**1.3**	0.2	**0.7**	0.2	**0.8**	0.2	**0.8**	0.1	**0.3**	0.0[aa]
PC 36:5	**0.3**	0.0	**0.3**	0.0	**0.4**	0.1	**0.4**	0.0	**0.5**	0.1
PC 36:4	**1.8**	0.3	**3.5**	1.0	**5.6**	1.1[a]	**4.9**	0.7	**5.7**	0.7
PC 36:3	**2.6**	0.3	**4.1**	0.7	**4.5**	0.6[a]	**4.3**	0.4	**5.6**	0.4[aa]
PC 36:2	**9.2**	0.4	**10.9**	1.2	**10.6**	0.4	**11.9**	0.6[a]	**13.3**	1.0[aa,b]
PC 36:1	**10.1**	0.5	**7.3**	1.3	**7.1**	1.1	**6.4**	0.7[a]	**4.0**	0.6[aaa]
PC 36:0	**5.0**	0.2	**3.6**	0.8	**3.0**	0.5[a]	**2.9**	0.5[a]	**3.2**	0.6
PC 38:6	**1.9**	0.1	**2.6**	0.6	**2.6**	0.4	**2.2**	0.2	**2.7**	0.3
PC 38:5	**1.6**	0.1	**1.7**	0.2	**1.9**	0.2	**1.6**	0.1	**1.8**	0.2
PC 38:4	**1.5**	0.2	**2.7**	0.6	**4.3**	0.9[a]	**3.9**	0.6	**4.8**	0.7[a]
PC 38:3	**1.3**	0.1	**1.9**	0.1	**2.1**	0.2[a]	**2.0**	0.2[a]	**2.6**	0.2[aaa]
PC 38:2	**1.6**	0.1	**1.7**	0.4	**1.5**	0.1	**1.5**	0.1	**2.0**	0.1
PC 38:1	**3.1**	0.2	**2.3**	0.5	**2.0**	0.4	**1.7**	0.3[aa]	**1.5**	0.2[a]
PC 40:6	**1.1**	0.1	**1.3**	0.1	**1.1**	0.1	**1.1**	0.1	**1.2**	0.1
PC 40:5	**0.7**	0.1	**0.7**	0.1	**0.5**	0.1	**0.5**	0.0	**0.6**	0.1
PC 40:4	**0.4**	0.0	**0.5**	0.1	**0.5**	0.1	**0.5**	0.1	**0.8**	0.1[aa]

**Phosphatidylethanolamine species**										
	Healthy		HP		AD		Neo		Her	
	Mean	SEM	Mean	SEM	Mean	SEM	Mean	SEM	Mean	SEM
PE 32:2	**1.3**	0.2	**1.5**	0.3	**0.8**	0.1[a,b]	**0.8**	0.1	**1.1**	0.1
PE 32:1	**1.6**	0.1	**1.5**	0.3	**1.0**	0.1[aa]	**1.1**	0.1[aa]	**1.2**	0.2
PE 32:0	**2.3**	0.2	**1.8**	0.4	**1.6**	0.2	**1.6**	0.2	**1.2**	0.2[a]
PE 34:3	**1.3**	0.1	**1.4**	0.3	**0.9**	0.1	**1.1**	0.1	**1.3**	0.2
PE 34:2	**4.4**	0.4	**4.3**	0.8	**4.5**	0.3	**4.8**	0.3	**4.2**	0.6
PE 34:1	**23.7**	3.5	**13.4**	5.5	**19.8**	3.8	**17.6**	2.8	**7.4**	1.5[a]
PE 34:0	**1.4**	0.2	**1.4**	0.5	**0.9**	0.1	**0.8**	0.1	**1.0**	0.1
PE 36:5	**0.9**	0.1	**1.0**	0.1	**0.7**	0.1	**0.7**	0.1	**1.0**	0.1
PE 36:4	**1.3**	0.1	**1.8**	0.3	**2.4**	0.2[aa]	**2.4**	0.2[aa]	**2.8**	0.1[aaa]
PE 36:3	**1.7**	0.1	**2.0**	0.2	**1.9**	0.1	**2.1**	0.1	**2.3**	0.2[a]
PE 36:2	**6.7**	0.4	**6.8**	1.7	**7.4**	0.4	**7.7**	0.4	**6.8**	0.9
PE 36:1	**7.3**	0.7	**6.0**	1.4	**6.2**	1.0	**6.5**	0.7	**3.5**	0.4[a]
PE 38:6	**2.7**	0.3	**4.1**	1.2	**5.2**	1.0	**4.9**	0.8	**6.0**	0.6
PE 38:5	**2.7**	0.3	**4.2**	0.6	**4.5**	0.7	**4.2**	0.4	**5.0**	0.3
PE 38:4	**5.5**	0.8	**9.1**	3.1	**13.5**	2.3	**14.5**	1.8[a]	**17.9**	2.6[aa]
PE 38:3	**2.9**	0.3	**3.8**	0.5	**2.6**	0.2	**2.7**	0.2	**3.5**	0.3
PE 38:2	**4.4**	0.2	**3.6**	0.5	**3.2**	0.3[a]	**3.3**	0.3	**3.2**	0.4
PE 38:1	**3.0**	0.2	**2.9**	0.4	**2.2**	0.2	**2.2**	0.1[a]	**2.4**	0.1
PE 40:6	**2.6**	0.6	**1.9**	1.3	**4.3**	0.8	**3.2**	0.6	**4.0**	0.7
PE 40:5	**3.0**	0.4	**4.1**	0.7	**2.7**	0.3	**3.0**	0.2	**4.3**	0.4[cc,d]
PE 40:4	**3.0**	0.3	**3.2**	0.4	**2.5**	0.2	**2.8**	0.2	**3.9**	0.4
PE 40:3	**3.2**	0.3	**3.6**	0.8	**2.1**	0.2	**2.5**	0.2	**3.1**	0.4
PE 42:7	**7.8**	0.9	**10.2**	2.6	**5.1**	0.6[b]	**5.7**	0.6[b]	**7.7**	0.7
PE 42:6	**3.0**	0.5	**3.0**	0.8	**2.0**	0.3	**1.9**	0.2	**2.7**	0.4
PE 42:5	**2.7**	0.3	**3.4**	0.8	**1.7**	0.2[b]	**2.0**	0.2	**2.4**	0.2

**Phosphatidylinositol species**										
	Healthy		HP		AD		Neo		Her	
	Mean	SEM	Mean	SEM	Mean	SEM	Mean	SEM	Mean	SEM
PI 32:0	**10.9**	0.8	**9.0**	2.0	**5.6**	0.8[aaa]	**6.6**	0.7[aa]	**5.1**	0.8[aaa]
PI 34:2	**6.8**	0.3	**5.2**	0.4	**4.8**	0.3[aaa]	**5.5**	0.3	**5.6**	0.5
PI 34:1	**14.0**	1.2	**10.8**	2.1	**8.9**	1.1[a]	**8.9**	0.9[aa]	**8.2**	1.3[a]
PI 36:4	**3.2**	0.6	**4.1**	0.5	**3.9**	0.2	**3.5**	0.3	**3.7**	0.2
PI 36:3	**3.6**	0.5	**3.9**	1.2	**4.0**	0.3	**3.6**	0.2	**3.7**	0.4
PI 36:2	**15.5**	1.0	**16.2**	1.5	**15.8**	0.9	**18.3**	0.5	**19.0**	1.2
PI 36:1	**12.7**	0.7	**10.8**	1.5	**8.3**	1.0[a]	**8.9**	1.0[a]	**7.5**	0.5[a]
PI 36:0	**10.0**	0.8	**8.2**	1.6	**5.2**	0.8[aaa]	**6.6**	0.7[a]	**5.1**	0.6[aa]
PI 38:4	**16.1**	1.9	**23.0**	5.3	**33.6**	3.7[aa]	**30.1**	2.9[a]	**32.7**	3.4[a]
PI 38:3	**5.6**	0.5	**7.0**	0.8	**7.5**	0.5	**6.7**	0.6	**7.6**	0.6
PI 38:2	**1.5**	0.5	**1.8**	1.0	**2.4**	0.3	**1.3**	0.3	**1.7**	0.5
**Lysophophatidylcholine species**										
LPC 15:0	**0.7**	0.0	**0.7**	0.1	**0.6**	0.0	**0.6**	0.0	**0.6**	0.0
LPC 16:1	**1.2**	0.1	**1.3**	0.1	**1.3**	0.1	**1.2**	0.1	**1.3**	0.1
LPC 16:0	**57.1**	1.1	**55.6**	2.7	**51.0**	1.5[a]	**54.3**	0.7	**51.8**	1.9
LPC 18:3	**0.4**	0.0	**0.4**	0.0	**0.3**	0.0	**0.4**	0.0	**0.4**	0.0
LPC 18:2	**5.3**	0.7	**7.3**	2.1	**8.3**	1.1	**6.8**	0.5	**10.7**	1.5[aa]
LPC 18:1	**10.0**	0.6	**11.7**	1.5	**13.2**	0.8[aa]	**11.5**	0.5	**10.9**	0.5
LPC 18:0	**19.4**	0.9	**16.3**	2.2	**17.1**	1.2	**17.7**	0.7	**16.4**	0.9
LPC 20:5	**0.3**	0.0	**0.3**	0.0	**0.4**	0.0	**0.4**	0.0	**0.4**	0.1
LPC 20:4	**1.4**	0.2	**2.3**	0.6	**3.1**	0.5[a]	**2.9**	0.3	**3.1**	0.3
LPC 20:3	**0.5**	0.1	**0.8**	0.2	**0.9**	0.1[a]	**0.7**	0.1	**1.0**	0.1[aa]
LPC 20:0	**0.6**	0.0	**0.5**	0.0	**0.5**	0.1	**0.5**	0.1	**0.4**	0.0
LPC 22:6	**0.4**	0.1	**0.6**	0.1	**0.9**	0.2[a]	**0.7**	0.1	**0.8**	0.1
LPC 22:5	**0.2**	0.0	**0.3**	0.0	**0.3**	0.0	**0.3**	0.0	**0.3**	0.0
LPC 22:4	**0.2**	0.0	**0.3**	0.0	**0.3**	0.0	**0.3**	0.0	**0.3**	0.0
LPC 22:0	**2.2**	0.2	**1.7**	0.3	**1.7**	0.2	**1.7**	0.2	**1.4**	0.1

Sphingomyelin species										
	Healthy		HP		AD		Neo		Her	
	Mean	SEM	Mean	SEM	Mean	SEM	Mean	SEM	Mean	SEM
SM 32:1;O2	**3.8**	0.2	**4.2**	0.6	**3.7**	0.1	**3.3**	0.1	**3.8**	0.3
SM 33:1;O2	**1.9**	0.1	**2.1**	0.2	**1.8**	0.1	**1.8**	0.1	**1.9**	0.1
SM 34:2;O2	**3.1**	0.2	**3.3**	0.3	**3.3**	0.2	**3.2**	0.1	**3.6**	0.1
SM 34:1;O2	**38.4**	0.7	**40.1**	1.3	**37.1**	0.7	**39.6**	0.7	**37.3**	0.4
SM 34:0;O2	**1.9**	0.1	**1.6**	0.2	**1.6**	0.1	**1.8**	0.1	**1.4**	0.1[a]
SM 36:2;O2	**2.5**	0.2	**2.5**	0.3	**2.5**	0.2	**2.5**	0.1	**2.7**	0.1
SM 36:1;O2	**7.5**	0.3	**7.4**	0.5	**7.1**	0.4	**7.5**	0.3	**7.2**	0.2
SM 36:0;O2	**0.5**	0.1	**0.4**	0.1	**0.4**	0.0	**0.6**	0.0	**0.4**	0.0
SM 38:2;O2	**1.1**	0.1	**1.1**	0.1	**1.1**	0.1	**1.0**	0.1	**1.3**	0.1
SM 38:1;O2	**3.4**	0.3	**3.2**	0.5	**3.2**	0.2	**3.0**	0.2	**3.8**	0.2
SM 40:3;O2	**0.1**	0.0	**0.2**	0.1	**0.1**	0.0	**0.1**	0.0	**0.2**	0.0
SM 40:2;O2	**3.5**	0.2	**3.8**	0.3	**4.2**	0.3	**3.6**	0.2	**5.0**	0.2[aa]
SM 40:1;O2	**8.2**	0.3	**7.5**	0.5	**8.0**	0.3	**7.5**	0.3	**8.2**	0.3
SM 40:0;O2	**0.1**	0.1	**0.0**	0.0	**0.1**	0.0	**0.1**	0.0	**0.0**	0.0
SM 42:4;O2	**0.1**	0.0	**0.2**	0.1	**0.3**	0.1	**0.2**	0.0	**0.4**	0.1[a]
SM 42:3;O2	**3.1**	0.2	**3.6**	0.7	**4.1**	0.5	**3.8**	0.3	**4.9**	0.3[a]
SM 42:2;O2	**12.0**	0.3	**12.0**	1.0	**12.8**	0.4	**12.9**	0.3	**11.8**	0.5
SM 42:1;O2	**8.8**	0.8	**6.8**	1.2	**8.6**	1.0	**7.5**	0.6	**6.1**	0.3
**Ceramide species**										
Cer 18:1;O2/16:0	**7.5**	0.4	**8.8**	0.5	**7.5**	0.3	**9.0**	0.5	**8.9**	0.5
Cer 18:1;O2/18:0	**4.8**	0.4	**5.3**	0.6	**4.2**	0.2	**5.0**	0.3	**5.1**	0.3
Cer 18:1;O2/20:0	**4.5**	0.5	**4.9**	0.6	**4.1**	0.3	**4.9**	0.3	**5.2**	0.2
Cer 18:1;O2/22:0	**13.5**	0.3	**13.3**	0.3	**12.9**	0.3	**12.8**	0.4	**13.4**	0.5
Cer 18:1;O2/23:0	**9.6**	1.1	**10.0**	0.9	**9.2**	0.9	**8.8**	0.6	**11.9**	0.4
Cer 18:1;O2/24:1	**29.6**	2.3	**26.7**	2.8	**30.3**	1.9	**31.0**	1.6	**21.7**	1.1[d]
Cer 18:1;O2/24:0	**30.6**	1.0	**31.1**	1.0	**31.9**	0.8	**28.4**	0.7[c]	**33.9**	1.0[ddd]

Values are expressed as a percentage of total membrane lipid (mole %) and represent the mean ± SEM, n=13 for healthy group, n=5 for HP group, n=16 for AD patients, n=9 for patients for Her patients, and n=19 for Neo patients. Statistical differences were assessed by one-way ANOVA followed by a Bonferroni post-test. a $p < 0.05$, aa $p < 0.01$, aaa $p < 0.001$, Primary vs HP, AD, Neo or Her; b $p < 0.05$, bb $p<0.01$, bbb $p< 0.001$, HP vs. AD, Neo or Her; c $p < 0.05$, cc $p< 0.01$, ccc $p < 0.001$, AD vs. Neo or Her; d $p < 0.05$, dd $p < 0.01$, ddd $p<0.001$, Neo vs. Her. Abbreviations: AD: adenomatous polyps; Cer: ceramide; Her: Hereditary CRC; HP: hyperplastic polyps; Neo: Neoplastic lesion (CRC); PC: phosphatidylcholine; PE: phosphatidylethanolamine; PI: phosphatidylinositol; SM: sphingomyelin.

Supplementary Table 4. Sensitivity and specificity values for the Σ34:1species to Σ38:4 species ratio

**Statistic**	**Value**	**95% CI**
Sensitivity	54.55%	38.85% to 69.61%
Specificity	94.44%	72.71% to 99.86%
Positive Likelihood Ratio	9.82	1.43 to 67.22
Negative Likelihood Ratio	0.48	0.34 to 0.68
Positive Predictive Value	96.00%	77.80% to 99.40%
Negative Predictive Value	45.95%	37.64% to 54.49%

For these calculations, we used the https://www.medcalc.org/calc/diagnostic_test.php webpage. The cut off value was established in 6.0. The comparison was established between benign and malign conditions.
